# Evaluation of a coaching workshop for the management of veterinary nursing students’ OSCE-associated test anxiety

**DOI:** 10.1186/s13620-018-0127-z

**Published:** 2018-07-27

**Authors:** Karen Dunne, Jenny Moffett, Sinead T. Loughran, Vivienne Duggan, Deirdre P. Campion

**Affiliations:** 1 Department of Applied Sciences, Dundalk Institute of Technology, Dublin Road, Dundalk, Ireland; 2 HPEC, RCSI, 123 St Stephen’s Green, Dublin 2, D02 YN77 Ireland; 3 School of Veterinary Medicine, Veterinary Science Centre, Belfield, Dublin 4, Ireland

**Keywords:** Test anxiety, Veterinary nurse, Objective structured clinical examination, Personality, Stress

## Abstract

**Background:**

High stress levels amongst undergraduates (particularly in relation to assessment) and efforts to improve mental wellbeing have been increasingly reported in the veterinary educational literature. However reports to date have primarily focused on the experiences of students of veterinary medicine, rather than veterinary nursing students.

**Methods:**

The purpose of this mixed method sequential explanatory study was to establish the “Big-five” personality traits and quantify the level of test anxiety associated with objective structured clinical examinations (OSCEs) amongst a cohort of 23 final year veterinary nursing students at an Irish third level college. The 12 item Brief FRIEDBEN Test Anxiety Scale (B-FTAS) and the 20 item mini International Personality Item Pool (mini-IPIP) were used to identify test anxiety levels and personality traits in this cohort. Focus groups were then employed to examine the effectiveness of a coaching intervention in ameliorating this test anxiety.

**Results:**

The initial, quantitative, phase found these students to have higher levels of test anxiety than previously reported for undergraduates sitting written examinations. No association was found between test anxiety and neurotic personality traits in this student cohort. In the qualitative follow up phase the coaching intervention was reported to have been helpful in equipping the students to better manage test anxiety. The OSCE stressors identified in this study closely resembled those previously reported by nursing and midwifery students.

**Conclusions:**

The shared experience of the coaching intervention and formative OSCE was reported to have been helpful in empowering the students to manage assessment-associated anxiety. Implications and recommendations for educators were identified.

**Electronic supplementary material:**

The online version of this article (10.1186/s13620-018-0127-z) contains supplementary material, which is available to authorized users.

## Background

### Stress in veterinary training and assessment

Training and working in the veterinary professions is recognized as being potentially highly stressful [1–7]. Stress levels during veterinary undergraduate training have been linked to high workload and assessment burdens [8]. This is mirrored in the medical nursing and midwifery literature, where both educators and student nurses report performance-based competency assessments to be potentially stressful experiences [9–12]. The importance of mental wellbeing and efforts to improve it are increasingly reported in the recent veterinary educational literature, particularly in relation to veterinary medicine undergraduates [13–16]. However there is currently an absence of reports in the literature examining the wellbeing and assessment experiences of veterinary nursing students.

Objective structured clinical examinations (OSCEs) are frequently employed to objectively test students’ capabilities against a criterion-referenced competent performance standard [17–22]. Students must demonstrate safe or acceptable levels of performance in order to graduate, register or progress to the next stage of training [23–27]. There is general agreement in the medical educational literature that to achieve competence learners must integrate knowledge, psychomotor skills and affective emotions/attitudes to perform clinical tasks appropriately [28–34].

Khan and Ramachandran [32] include stress-induced cognitive dysfunction as a factor that may reduce performance by reducing clarity of thought. In addition, they point out that while students can learn the knowledge, attitudes and psychomotor skills needed to improve performance, it is more difficult for them to acquire the ability to maintain performance while anxious, tired, under observation or in a high-pressure situation, such as working on a critically ill patient. These conditions could equally apply to high-stakes summative performance assessments, such as an OSCE, where they may also limit performance [9]. OSCEs have been previously reported to induce high levels of test anxiety amongst students [27, 35–37].

Al Ghareeb et al. [12] note that while the terms “stress” and “anxiety” are often used interchangeably their sources are different. Stress arises due to external pressure while anxiety is an internal cognitive response. Test anxiety is a subjective feeling of apprehension prior to and during an assessment. Von der Embse and Witmer [38] found test anxiety to be negatively correlated with performance. Some test anxiety is necessary to motivate students to prepare for examinations but it can be argued that failure to perform competently on the day due to anxiety-induced cognitive dysfunction reduces the validity of the assessment and is, thus, wasteful of resources. Running an OSCE is recognized as being heavily resource-intensive [22, 23, 39, 40]. Holding repeat sittings for candidates, who may actually already be competent but fail the assessment due to anxiety, costs both time and money. Furthermore it may be a negative and stressful experience for the students involved [41].

### Personality traits and stress

The “Big Five” personality theory is a widely used model that identifies five major personality traits [42] (Table 1). The interplay between these traits and a variety of other factors, such as environment, culture and society, influence how an individual experiences life. Links have been drawn in the educational literature between neuroticism and issues with learner self-regulation and emotional stability [43, 44]. High neuroticism has also been linked to reduced levels of personal wellbeing and elevated stress levels [45–48]. Studies of the nursing profession have found high neuroticism to be associated with emotional exhaustion [49], depersonalization [50] and secondary traumatic stress, leading to increased risk of burnout [51]. A recent survey of UK-based veterinarians found neuroticism to be more strongly associated with occupational stress in this population than environmental factors such as workload [52].

**Table 1:** Big Five personality traits and associated characteristics (adapted from Dawson and Thompson, 2017)

Trait	Characteristics
Extraversion	Warmth, gregariousness, assertiveness, activity, excitement seeking, positive emotions
Agreeableness	Trust, straightforwardness, altruism, compliance, modesty, tender-mindedness
Conscientiousness	Competence, order, dutifulness, achievement striving, self-discipline, deliberation
Neuroticism	Anxiety, anger, hostility, depression, self-consciousness, impulsiveness, vulnerability
Openness	Fantasy, aesthetics, feelings, actions, ideas, values

### Study aims

There is a current lack of published data pertaining to the personality traits of veterinary nursing students, the levels of OSCE-associated test anxiety they may experience and guidance for educators and students in how to effectively reduce or manage this anxiety. It is this literature gap that this preliminary study is intended to address.

This article reports on a study conducted to examine the personality traits and quantify the levels of test anxiety associated with OSCE assessment amongst a group of veterinary nursing students. It also explores the effectiveness of a coaching intervention intended to assist with managing this anxiety.

### Study design

**Fig. 1:**
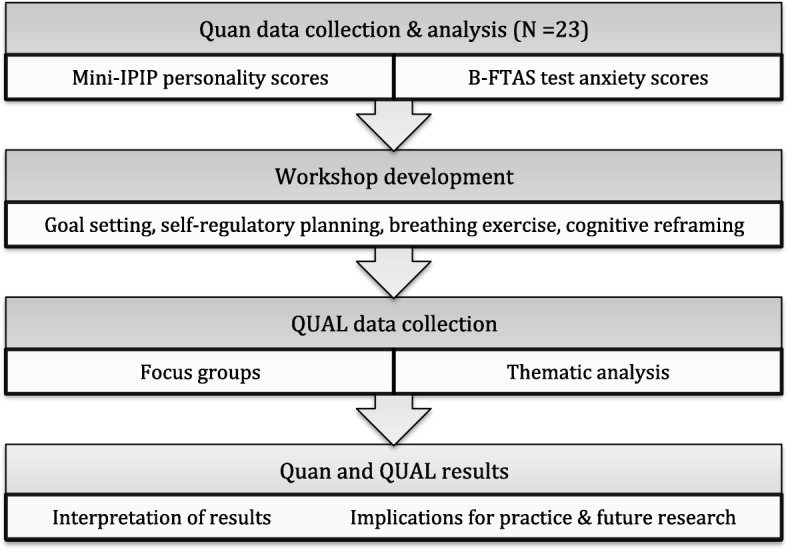
Study design: mixed method sequential explanatory study (with qualitative priority)

A mixed method sequential explanatory study (with qualitative priority) was used (Fig. 1) [53], based on a pragmatic philosophical rationale that the combination of these methods would allow for a more complete picture to be obtained of the research problem [54]. The initial, quantitative, phase used validated survey instruments [55, 56] to record the Big-Five personality attributes and levels of test anxiety reported by a class of 23 final year veterinary nursing undergraduate students at Dundalk Institute of Technology (DkIT), Ireland.

The second, qualitative, phase used focus groups to obtain deeper insights into the students’ experiences of the intervention and explain its effects [57]. The effectiveness of a coaching workshop designed to ameliorate OSCE anxiety was examined. This study was intended as a preliminary exploration of test anxiety, and efforts to manage it in order to inform future practice during OSCE-based competency assessment in veterinary nursing education.

## Methods

### Background

The B.Sc. in Veterinary Nursing at DkIT is an ordinary level degree course of three years in duration. To be eligible for registration as a veterinary nurse, students are required by the national veterinary regulator, the Veterinary Council of Ireland (VCI), to pass a summative competency assessment (blueprinted to the European veterinary nursing competencies [58]) in the form of an eight-station OSCE prior to graduation.

### Quantitative data collection

In May 2017 all 23 students in the third year veterinary nursing cohort were invited to take part in a workshop on managing OSCE test anxiety. The workshop was scheduled for the last week of term, one week before a formative “mock OSCE” (consisting of four stations) and one month before the final summative eight-station OSCE. Participants were briefed on the project by author one during a scheduled lecture period two weeks prior to the workshop and given hard copy questionnaires of the brief-FRIEDBEN test anxiety scale (B-FTAS) (Additional file 1) and a mini International Personality Item Pool (mini-IPIP) personality inventory (Additional file 2). Participants were asked to complete the B-FTAS specifically in relation to OSCEs, rather than examinations in general.

The B-FTAS questionnaire has previously been validated for use as a test anxiety screening tool [56]. It consists of 12 items across three subscales: five items on social derogation (SD), four on cognitive obstruction (CO) and three on physiological tenseness (PT) (Table 2). SD quantifies the level of anxiety caused by social concerns. CO measures the effect of anxiety on cognitive processes such as memory and recall, while PT captures the physical symptoms associated with test anxiety. A six-point Likert-type scale from one (*does not describe me at all*) to six (*describes me perfectly*), allows the results to be summed to give a total test anxiety score in the range 12–72.

**Table 2:** B-FTAS items

Subscale	Item	Text
Social derogation (SO)	1	If I fail a test, I am afraid what my friends will think.
	2	If I fail a test, I am afraid people will consider me worthless.
	3	I am very worried about what my teacher will think or do if I fail this test.
	4	I am worried that all my friends will get high scores on the test and only I will get low ones.
	5	I am worried that failure on the test will embarrass me socially.
Cognitive obstruction (CO)	6	During a test, my thoughts are clear and I answer all questions. (R)
	7	During a test, I feel that I’m in good shape and I’m organized. (R)
	8	I feel that my chances are good to perform well on tests. (R)
	9	I usually function well on tests. (R)
Physiological tenseness (PT)	10	I am very tense before a test, even if I am well prepared.
	11	While I am taking an important test, my heart beats rapidly.
	12	I am terribly scared of tests.

R = reverse scored item

The mini-IPIP scale is a shortened (20 item, four items per dimension) version of the IPIP Five-Factor Model measure. It facilitates valid and reliable measurement of the Big Five factors of personality on a 5-point Likert-type scale ranging from one (*very inaccurate*) to five (*very accurate*), to give a score in the range 4–20 for each trait (Table 3) [45, 47, 55].

**Table 3:** Mini-IPIP items (adapted from Donnellan et al. 2006)

Item	Factor	Text
1	E	I am the life of the party
2	A	I sympathise with others’ feelings
3	C	I get chores done right away
4	N	I have frequent mood swings
5	O	I have a vivid imagination
6	E	I don’t talk a lot (R)
7	A	I am not interested in other peoples’ problems (R)
8	C	I often forget to put things back in their proper place (R)
9	N	I am relaxed most of the time (R)
10	O	I am not interested in abstract ideas (R)
11	E	I talk to a lot of different people at parties
12	A	I feel others’ emotions
13	C	I like order
14	N	I get upset easily
15	O	I have difficulty understanding abstract ideas (R)
16	E	I keep in the background (R)
17	A	I am not really interested in others (R)
18	C	I make a mess of things (R)
19	N	I seldom feel blue (R)
20	O	I do not have a good imagination (R)

*E* extraversion; *A* agreeableness; *C* conscientiousness; *N* neuroticism; *O* openness (sometimes also referred to as intellect/imagination); *(R)* reverse scored item

### Qualitative data collection

The workshop was delivered by author two and consisted of a short presentation on test anxiety and coping skills, followed by a number of interactive small group exercises which supported learning on self-regulatory planning, breathing for relaxation, and cognitive reframing. It was followed by the “mock OSCE” one week later. Measures intended to reduce student anxiety were incorporated into the examination waiting area. These consisted of an informal group seating arrangement, positive affirmation posters and relaxing background music^1^ played on a loop.

A purposeful typical sampling strategy [59] was used: all the workshop attendees completed the “mock OSCE” and were sitting their final OSCE for the first time. All the workshop participants were therefore invited to participate in the focus groups to appraise the effectiveness of the intervention and to make suggestions for further improvement of the student experience. Two focus groups were held: one after each “mock OSCE” session (morning and afternoon). This avoided students who wished to participate having to wait around after the morning session. It also ensured that all students who wished to contribute had the opportunity to do so without the group exceeding a manageable size [54].

Two students (both female) attended the first focus group and three (two females, one male) attended the second. Each session lasted 30–45 min and was audio-recorded. Author two moderated the first session. Author three attended the first focus group as an observer and moderated the second. Neither moderator was involved in teaching or assessment on the veterinary nursing course. The same room, seating arrangement, interview guide and semi-structured questions (Additional file 3) were used in both focus groups, to ensure consistency between the two sessions.

### Quantitative data analysis

**Fig. 2:**
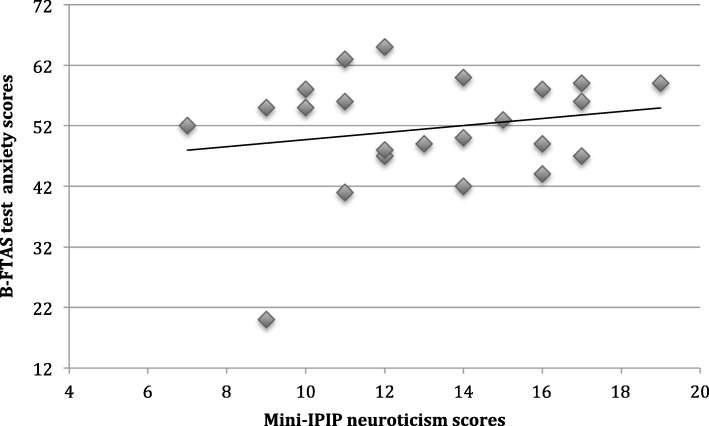
Neuroticism and test anxiety scores

The data from the hardcopy B-FTAS and mini-IPIP questionnaires that had been completed by the students was anonymised and entered into Microsoft Excel^2^ by author one. This enabled the participant test anxiety and mini-IPIP scores to be generated. Scores were plotted on a scatter graph to visualize the relationship between the variables (Fig. 2).

Spearman’s rank-order correlation coefficient was calculated to determine the strength and direction of association between total test anxiety scores, as measured by the B-FTAS questionnaire, and neuroticism, as measured by the mini-IPIP scale.

### Qualitative data analysis

Author one transcribed both focus group audio recordings. All names and identifying features were removed to maintain participant anonymity. The transcripts were then reviewed and verified by the participants. Thematic analysis [60] was performed by authors one and two. Microsoft Excel was used to manage and facilitate this process, as described by Bree and Gallagher [61]. A coding framework was developed and used to identify initial themes within the data across both focus group discussions. The data then underwent several rounds of review and refinement to identify the main themes and associated subthemes (Table 4). Each reviewer coded and themed the transcripts separately and the results were then compared and merged by consensus.

**Table 4:** Qualitative data analysis overview

Data analysis steps performed	Thematic analysis phase (Braun & Clarke, 2006)
Focus groups audio recorded	
Discussions transcribed in Microsoft Word^a^, anonymised and transferred to Microsoft Excel	1
Transcripts verified by participants to confirm their accuracy	1 & 2
Initial read through the data centred on the identification of themes	2 & 3
Themes were initially coded by cell colour to match thematic areas	3, 4 & 5
Microsoft Excel’s filter applied to sort the data by cell colour (grouping codes into thematic areas)	3, 4 & 5
Second pass over data identified overlaps and consolidated data points	4 & 5
Numerous further passes over data, condensing data at each stage to collapse codes into themes	5 & 6
Generation of data overview summary with key points under the three main themes which emerged	6

^a^Microsoft Word for Mac 2011, version 14.4.1, Microsoft Corporation, Redmond, WA

## Results

### Quantitative findings

All 23 students (21 females, 2 males) with a mean age of 24 years (*SD* = 5.9) completed and returned the questionnaires. Descriptive statistics, including the mean, standard deviation and minimum and maximum values, were used to describe the B-FTAS (Table 5) and mini-IPIP results (Table 6).

**Table 5:** B-FTAS results

Variable	Mean (*SD*)	Range
Social derogation (SD) (range 5–30)	18.52 (5.52)	5–28
Cognitive disruption (CD) (range 4–24)	17.17 (3.60)	10–23
Physiological tenseness (PT) (range 3–18)	15.87 (2.96)	5–18
Total B-FTAS score (range 12–72)	51.57 (9.46)	20–65

**Table 6:** Mini-IPIP results

Variable (range 4–20)	Mean (SD)	Range
Extraversion	10.35 (3.77)	4–17
Conscientiousness	13.87 (2.93)	9–19
Openness	14.61 (3.46)	7–20
Agreeableness	17.57 (2.52)	12–20
Neuroticism	13.13 (3.15)	7–19

No association was observed between test anxiety and neuroticism scores using the Spearman rho correlation coefficient (r_s_ = 0.115, *p* = 0.602). We concluded that test anxiety and neuroticism scores were not linked in this student cohort.

### Qualitative findings

The thematic analysis resulted in the identification of three themes and 30 subthemes (Table 7).

**Table 7:** Focus group themes and subthemes

Themes	Subthemes
1. Student experiences of OSCEs	Negative perceptions of OSCEs
	Preparation challenges
	Waiting time challenges
	OSCEs associated with high test anxiety
	Being “under scrutiny”
	Negative effects on performance
	Formative “mock OSCE” is helpful
	Staff support is helpful
	Memorization is a factor in preparation
2. Student experiences of the intervention	Empowerment
	Sense of perspective
	Value of sharing anxiety experiences with others
	Neutrality towards the intervention
	Positivity towards breathing exercises
	Positivity towards cognitive restructuring exercise (“OSCE animal”)
	Positivity towards waiting area alterations
	Waiting area alterations acted as a reminder
	Talking during waiting period
	Preference for waiting in small groups
	Negativity towards looped music while waiting
	Other helpful effects
3. Suggestions for future OSCE management	Suggestions related to the intervention
	Suggestions related to waiting room
	Desire for repeated practice opportunities
	Desire for additional resources to practice specific tasks/areas
	Attention to “real-life” details
	Replace OSCEs altogether
	More clarity on marking methodology
	Persistent fear despite interventions

### Theme one: Student experience of OSCEs

The focus group participants were in agreement that the OSCE was associated with significant anxiety and that this began from early in their training, often due to contact with more senior students.

*I’ve been freaking out about OSCEs from the first day of being a veterinary nurse! It’s the word “OSCEs”. It strikes terror into everyone’s heart I think, from day one…Just you hear the word OSCE and you’re like what’s that? And then they tell you, it’s…this practical exam where you have six minutes and you have to do everything perfectly and the lecturer is standing there staring over your shoulder. (Student 5)*

The students reported preparing for OSCEs differently from other exams. They recognized that theoretical knowledge alone was not sufficient but lack of access to equipment while revising was challenging. *“I find it harder to study for OSCEs because you’re trying to visualise everything because you don’t have the equipment there in front of you”* (Student 2).

Anxiety levels were reported as increasing significantly on the day of an examination and while waiting to start. *“I was driving in here on the way in from [home] this morning and I started to panic!”* (Student 3). *“But it’s just the waiting around and the dread and the overthinking everything before you go in…is the hard part I think”* (Student 1).

Waiting between stations was also highlighted as an anxious period, with the participants reporting a preference for minimal delays between the tasks.

*It was the waiting outside of the other rooms where you actually got to think about it and you kinda [sic] started to panic thinking about it. So...but once you’re in there doing the thing everything else is...it’s fine...but it’s the waiting that’s the worst part to me anyway. All of the waiting! (Student 2)*

*I went to the next [station] and it was taking a while and I was probably five minutes waiting outside and I was like ‘oh my God get me in!’ Just like get me in and started. Because the other two were bang, bang, no time to think, it was so much easier just to get into them and get started. (Student 4)*

The students reported anxiety as manifesting itself in a variety of ways during an OSCE. Both physiological tension (shaking) and cognitive obstruction (lack of clarity of thought) were described and these were recognized as having a negative effect on performance. *“Even though I didn’t really feel the nerves in my head, my hands were shaking…I was pipetting stuff and I couldn’t coordinate”* (Student 3).

*I have trouble, am, thinking what the next steps are and I could learn everything off…and still get there and not remember what I’m supposed be doing. (Student 1)*

The participants reported finding OSCEs as more challenging than other forms of assessment. Reasons for this included the time pressure and the fact that they were being observed.

*I have no problem doing practical or written exams. I know I have to work hard to do them but I can do them fine. It’s these [OSCEs], when someone is watching you and you feel like you’re on the spot...and I just don’t deal with it very well. (Student 1)*

Furthermore, once participants had experienced anxiety during a station they reported a tendency to carry this with them and it had a knock-on effect of reducing their performance in subsequent stations. *“I knew that I had completely messed it up so it kind of just puts you in a [negative] headspace for the next one then”* (Student 4).

However, participants acknowledged that these negative factors could be mitigated in a variety of ways. Completing this formative OSCE, whilst stressful, was also felt to be a valuable learning opportunity. *“It was a good learning experience…and even, kind of the logistics of it…we’ve never done four in a row before so that was good experience”* (Student 1). The DkIT veterinary nursing assessment strategy also incorporates low-stakes assessment of individual OSCE tasks into the various modules in which these skills are initially taught. These were also felt to be useful in preparing for the final OSCE. *“The practice OSCEs that we do kind of throughout the year…they really help”* (Student 4).

Participants reported pressurizing themselves to perform the tasks correctly, as they didn’t want to embarrass themselves by making errors in front of academic staff members who had taught them the procedures and were now evaluating their performance.

*Yeah I think it doesn’t help the fact that we know these lecturers as well and that just adds to the nerves and that’s because you feel embarrassed if you do anything wrong. I know you shouldn’t but you can’t really help it sometimes. (Student 2)*

However the conduct of these staff members during the assessment was perceived as supportive.

*The lecturers were lovely today! You know, if they see that you’re struggling or panicking they...they’re very helpful, you know, as far as they can be. (Student 1)*

### Theme two: Student experiences of the intervention

There was a positive opinion of the overall value of the coaching workshop. Participants reported feeling empowered and having gained a sense of perspective afterwards. *“[Lecturer X] and [workshop leader] pointed out that we’ve come this far. We can do it”* (Student 1). *“I just felt a bit calmer. It puts things more into perspective I suppose”* (Student 2). *“Even if you fail [the final OSCE] it’s not the end of the world”* (Student 5). This altered perspective improved participants’ abilities to deal with mistakes and uncertainty as they arose during the formative OSCE.

*The workshop reinforced when you do one: let it go. That’s it. Done. Gone…I think the workshop really helped me to just separate [an OSCE task] down into those constituent parts, and just do one, move on, do the next one and just focus on what I’m doing instead of worrying about [the previous step]. (Student 5)*

The shared experience of sitting in the room and hearing other students and the workshop leader talk openly about the assessment anxiety they felt and how they tried to manage it was reported to be very valuable. *“The workshop [was helpful] just to kind of realise that everyone is in the same boat. I think that really helped”* (Student 1).

*I liked that…every point [the workshop leader] brought up…about how you feel coming up to exams, it was like ‘everyone feels that way’. It kind of made you realise that you’re not the only person here who feels that they’re not good enough or feels that ‘oh everyone else is studying and they know more than me’, this kind of thing. She was bringing up all these points and it was ‘oh God, yeah, that’s exactly how I feel’ and…she was saying herself that she feels that way, you know, before exams and if a vet [emphasis] who is, you know, doing this for a living can feel that way then you kind of feel better about yourself. (Student 4)*

With regards to the content of the workshop, some participants spoke favorably about the breathing techniques and reported them to be beneficial. In addition, there was consensus as to the benefits of the cognitive reframing exercise, which had involved participants visualizing their “exam animal” and thinking about how they could better prepare it for the upcoming assessment.

*I think the animal stayed with me and the fact that [the workshop leader] said that we can, you know, care or for tend the animal and they can change. Even though I know I said that I have doubts at my stage of life that I can! I did like that idea. I think that did resonate with me. That did stick with me, yeah. (Student 1)*

Finally, other workshop benefits that were identified included insights into the biology of stress and the potential for humour to be engaged as a coping strategy.

The changes that had been introduced to the OSCE waiting area were also generally perceived as positive and conducive to a more relaxed atmosphere. Most of the participants agreed that a small group (4–5 people) was preferable while waiting.

*I think it’s just that, if there was a larger group, people would tend to clump into smaller groups around the room and then you’ve got four or five different conversations going on and you’re just kind of listening to everything and not hearing anything. Whereas when there were four of us there today...we were just talking, like, do you know? (Student 5)*

Having the opportunity to chat amongst the small group while waiting was also beneficial. However the participants were aware that not everyone else might feel the same way and were mindful of others’ preferences.

*I feel like I come in and I have to chat, just to take my mind off things, which is probably not helpful for everyone else though! [laughter] Am, I ah, yeah I find the talking kind of helps my nerves but, it probably doesn’t help anyone else so I probably need to keep an eye on that for the real [OSCE]! (Student 1)*

The looped music was the only negative feature of the waiting area to be raised.

*The one thing that drove me mad was the music! [agreement from others]…I can see how that would work for other people but just for me personally I just, I can’t...[trails off]. (Student 5)*

### Theme three: Suggestions for future OSCE management

The focus group participants were in agreement that the workshop was of value and recommended that it be continued. The participants also agreed that the waiting room changes should be retained and recommendations here related to maintaining small group sizes and alternatives to the background music: either having none or facilitating the use of headphones.

The small groups were also reported as contributing to a positive mindset amongst the participants and this was another reason put forward for their retention.

*And then you kind of all go out together and it’s like ‘just do this’, like a team! [laughs]. “Yeah, yes” [other students agree]. As you were coming out of one someone else would be going in and you were kind of like “yeah go!”...kind of encouragement like. (Student 4)*

Other suggestions related to OSCE preparation, specifically practice opportunities in the lead up to the final assessment. Participants reported independent skills practice at home, in their workplace (if they were employed part time in a veterinary practice) and in the college skills laboratory. *“Yes like our bandaging we can go home and practice on our own dogs or teddies or whatever”* (Student 3). However the main drawback of this was the lack of access to some equipment that would be in use in the final OSCE. Additional exposure to this specific equipment, such as the anaesthetic machine, was felt to be desirable.

*But then things like the anaesthetic machine...we only get to see it during those couple of practicals so sometimes, you know, if you have one in work it’s grand but it might be completely different to the one in here. (Student 4)*

An additional revision session towards the end of the semester but before the written examinations was requested. Some participants requested that lecturers be present for guidance whilst others felt that access to the equipment alone would be sufficient.

Directly observed procedural skills (DOPS) were mentioned as an alternative to OSCEs. The students had some experience of these but preferred the OSCE format, as they were not required to explain their reasoning during the tasks. This led to a discussion about talking during the OSCE and whether it was helpful or not. Some participants felt that this could help to dissipate anxiety whilst others worried that it could be counter-productive if the student inadvertently revealed a lack of knowledge or understanding about the task they were performing and there was a desire for further clarification on the marking methodology.

*If you kind of think that you’re getting marked on your OSCE as well as getting marked on what you’re saying, you’re like ‘oh God, what if I say something wrong? Are they going to fail me on that?’ Rather than…just what I’m doing. (Student 4)*

The participants concluded by stating that whilst steps to try and mitigate anxiety were helpful and should be continued, the final summative OSCE is ultimately a high-stakes examination and the anxiety associated with it would always persist at some level.

*Yeah I think I’m going to be always dreadfully nervous about these kinds of things [other student “yes, yes”]. Am and, I don’t know if there’s a way that I can’t not be nervous! [laughs]. (Student 1)*

## Discussion

### OSCEs as a stressful experience

Our findings confirmed that this cohort of veterinary nursing students did experience OSCE-associated test anxiety. This anxiety was present from early in their training and the stressors identified in this study resemble those reported by nursing and midwifery students [9, 35]. Awareness of these stressors is useful for educators, as it enables measures to be implemented to address them. This study did not find high neuroticism and test anxiety levels to be linked in this cohort of students. It is likely that an OSCE is an inherently stress-inducing experience. Educators should consider interventions to mitigate this stress where possible, as it is likely to affect all candidates to some extent, and not just those individuals with personality traits conducive to higher anxiety levels.

The total B-FTAS scores (M = 51.57, *SD* = 9.46) of this student cohort are higher than those previously reported by undergraduates sitting written examinations. A previous study tested 487 undergraduate students under both fear-associated and efficacy-associated conditions and reported mean total B-FTAS scores of 35.46 (*SD* = 13.20) and 36.13 (*SD* = 10.27) respectively [62]. The numbers involved in this study are too small to draw any direct conclusions about anxiety levels amongst veterinary nursing students in general. Nevertheless they are an initial attempt to quantify the generally accepted, but hitherto mostly anecdotal perception, that veterinary nursing students do experience high levels of test anxiety associated with a final summative OSCE.

High levels of test anxiety, particularly on the CD and PT subscales, have been associated with reduced test performance [38]. When viewed from the perspective of van der Vleuten’s assessment utility equation [63], high test anxiety has the potential to lower the overall utility of an OSCE as an assessment of competence, due to its negative effects on validity, reliability and context [64].

The Big Five personality traits co-exist but are each present to varying degrees in each individual, giving rise to a range of personalities [65]. Conscientiousness and agreeableness are positively associated with self-efficacy but high neuroticism levels have a negative impact on both it and resilience [48] and have been associated with an increased incidence of compassion fatigue and secondary traumatic stress in nurses [66].

A recent study recorded mini-IPIP scores for 4292 college students and reported a mean neuroticism score of 10.51 (*SD* = 3.01) in this population [46]. The mean neuroticism score reported in the current study is higher (M = 13.13, *SD* = 3.15) but the small number of participants precludes inferences being drawn about neuroticism levels amongst veterinary nursing students in general. Nevertheless, personality factors and anxiety levels amongst veterinary nursing undergraduates have not previously been reported, so this represents a preliminary step to address this gap in the literature. Not all the students in this study had high neuroticism scores but the workshop was specifically designed to target those individuals who did, as this is the personality trait most associated with test anxiety [46].

### Similarities with nursing and midwifery student experiences

The veterinary nursing students in this study viewed OSCEs as challenging and stressful and these opinions developed very early in their training. These findings mirror the views previously expressed by nursing and midwifery students [9, 35] and are also in agreement with educators’ perceptions of summative OSCEs as an anxiety-inducing assessment [10, 24, 27, 37, 40]. The results of the current study add the “student voice” to the perspectives on stress levels and assessment anxiety present in the current veterinary educational literature.

The challenges posed by OSCE preparation were categorized by the students in this study as different to and more challenging than those encountered with other forms of assessment, such as written examinations. Performance under direct scrutiny, equipment concerns, worries that a previous task had been performed incorrectly and waiting before and between stations all contributed to the high anxiety levels associated with an OSCE. Studies of nursing and midwifery students reported strikingly similar concerns about OSCEs [9, 35, 67].

### Implications for future assessment practices

The qualitative findings in this study suggest insights into the reasons for the high test anxiety scores reported by these participants. The explanations voiced by the participants in this study are of value, as once educators are aware of particular stressors (such as excessive waiting, large group sizes etc.) steps may be taken to mitigate at least some of them [37]. The efforts made in this study to reduce stress were very straightforward to implement e.g. the provision of informal seating and motivational posters in the waiting areas.

Participation in the workshop benefitted participants both by equipping them with additional tools to manage anxiety and improving their perceived self-efficacy in dealing with stressful situations. Workshop participation was reported to be empowering, as it provided the participants with additional strategies to manage anxiety. This resembles previous reviews of the coping strategies employed by college students in which student motivation and course grades were positively associated with problem-focused coping (where the subject feels empowered to utilise strategies intended to diminish the stressful effects of an event), but not emotion-focused coping (where individuals perceive a stressful event as something that must be endured and seek to reduce or avoid the negative emotions associated with it) [7, 41, 68].

Attending the workshop was found to be a valuable experience; hearing others talk about their struggles with exam anxiety was highlighted by the participants as particularly useful. One student reported how she had previously felt that everyone else coped with exam pressure much more readily than she did. However the workshop discussion revealed that the vast majority of her peers also felt anxious about assessment and this insight boosted her self-confidence.

Participants also noted that the workshop had helped them to develop a better sense of perspective about the OSCEs. If they made a mistake they felt better able “to let it go” and switch their focus to the next task, rather than viewing an error as a catastrophe that confirmed their incompetence. These findings reinforced the value of the workshop as a face-to-face session involving group exercises, as the shared experience of taking part in it empowered the students in a way that merely informing them about stress management strategies may not have achieved. Moffett and Bartram have noted that the ability to change one’s perspective has recently been linked to resilience [15]. Having a “growth” rather than a “fixed” mindset fosters a view of difficulty as an opportunity to learn and improve, not a confirmation of one’s lack of ability [69, 70]. Personality traits tend to be stable but mindsets relate to a person’s belief system and so can be altered; it is possible to move towards a growth mindset regardless of personality type [71].

There was general agreement amongst the participants that both the workshop and the changes made to the waiting area should be retained for future cohorts. There was however variation as to which strategies from the workshop the participants used and how they employed them. For example some found the breathing exercise beneficial whilst others did not use them. This finding supports the inclusion of a range of material in the workshop, thereby enabling individuals to select those most relevant to their own needs.

The students in this study expressed a desire for repeated practice opportunities, ideally on the same equipment that would be used in the final examination. This echoes the report by Jay who noted seemingly minor inconsistencies in equipment provision as a major source of anxiety for midwifery students in the high-pressure atmosphere of an OSCE [35]. In addition, some participants in this study wanted lecturers to be available for feedback/guidance during revision sessions. This contrasts with nursing and midwifery students who valued peer practice and feedback as helpful OSCE preparation [9, 35]. The reasons for this difference are unclear: it may be that the participants in this study felt that peer feedback might not adequately enable them to avoid errors. This is understandable, given the high-stakes nature of the assessment, but it is very difficult for academic staff to provide additional support during student revision sessions on top of their normal teaching commitments, especially in the current environment of limited resources and funding cutbacks in the Irish Higher Education sector [72].

Other suggestions for improvement related to group size while waiting before the assessment, with a preference expressed for dividing the assessment cohort into smaller groups (4–5 individuals). This finding also reflects the previously reported views of midwifery students, who found interactions with other students and waiting periods during an OSCE to be stressful [9, 35].

Finally, students disagreed with each other in relation to the benefits of talking whilst performing an OSCE task. Some individuals found it assisted them in keeping their composure whilst others were worried that it could raise doubts about their competency. This corroborates previous reports of the difficulties students may encounter with communication or dialogue during the stressful and simulated environment of an OSCE [9, 35, 40]. Jay recommends exploring alternative OSCE formats to allow for a more holistic assessment of competence, including communication skills [35].

### Limitations and recommendations for future research

A primary limitation of this study is the use of a single-group design to evaluate the effectiveness of the intervention. This was due to practical and ethical difficulties associated with the identification of an appropriate control group. The DkIT human research ethics committee could have viewed the workshop as a potentially beneficial teaching intervention that could not be legitimately withheld from one group of students. In addition, the student group involved is tight-knit, making efforts to prevent them informing excluded classmates about the intervention impossible to implement.

The focus groups were held after the formative “mock OSCE” rather than the final assessment. This was because the summative OSCE takes place during the final work placement component of the course and the students return to placement immediately after it. Therefore the participants would not have been available for a face-to-face evaluation following their final OSCE. Telephone interviews were considered but were rejected on the grounds that they could inconvenience the host practices and are not as natural an environment for open discourse as focus groups [73]. Hence it cannot be stated with certainty that this intervention would be effective in ameliorating the levels of test anxiety associated with a summative OSCE.

It is also possible that only students with a self-selection bias took part in the focus groups. Finally, this study reports only the experiences of a single cohort of students in one veterinary nursing course, making it difficult to generalize the findings. Further research could be conducted to measure test anxiety and neuroticism levels and to repeat the intervention with other/larger student cohorts, in addition to evaluating its effectiveness during summative assessments.

## Conclusions

The veterinary nursing students in this study reported significant levels of test anxiety, especially in relation to OSCEs. This anxiety is pervasive, present from an early stage of veterinary nursing training and appears to be independent of individual personality traits in this cohort. However the participants were aware of ways in which this anxiety could be addressed and further management strategies were obtained from the intervention reported here. These included additional coping skills and a shifting of perspective towards a greater sense of empowerment, self-belief and resilience. In particular, the experience of participating in the group exercises during the workshop session contributed to this perspective shift.

The authors suggest that educators consider group size and the waiting area environment when running practical assessments. The waiting area changes that were trialed this study, (background music, affirmative posters linked back to the workshop content and casual small group seating arrangements), were all low/no budget and very straightforward to implement. Given the close similarities between the concerns associated with OSCEs by the participants in this study and those of nursing and midwifery students in previous reports, these interventions could also be of value in the mitigation of test anxiety in students in these and other related disciplines, where similar assessment methods are commonly employed.

The aim of the interventions reported here was not to remove all anxiety (as some is inevitable and is indeed necessary for motivation to adequately prepare for examinations), but rather to introduce some simple and cost-effective stress-reduction practices in the staging of OSCEs and equip students with additional tools to better manage anxiety; thereby helping to prevent it from becoming a performance and validity-limiting factor during competence assessment.

## Additional files

Additional file 1: B-FTAS questionnaire. (PDF 78 kb)

Additional file 2: mini-IPIP questionnaires. (PDF 79 kb)

Additional file 3: focus group questions. (PDF 37 kb)

## Abbreviations

B-FTAS: Brief FRIEDBEN test anxiety scale
CO: cognitive obstruction
DkIT: Dundalk Institute of Technology
DOPS: Directly observed procedural skills
IPIP: International personality item pool
OSCE: Objective structured clinical examinations
PT: Physiological tenseness
RVN: Registered veterinary nurse
SD: Social derogation
VCI: Veterinary Council of Ireland

## Footnotes

1 Respiro (2016) by Mattia Vlad Morleo. Free download from Jamendo Music.

2 Microsoft Excel for Mac 2011, version 14.4.1, Microsoft Corporation, Redmond, WA.
