# Supplementary material for: Evaluation of a coaching workshop for the management of veterinary nursing students’ OSCE-associated test anxiety
Source: Ir Vet J. 2018 Jul 27;71:15. doi: 10.1186/s13620-018-0127-z (PMC6064137; doi:10.1186/s13620-018-0127-z)
Supplement: Supplementary file 1 — B-FTAS questionnaire. (PDF 78 kb) [file 13620_2018_127_MOESM1_ESM.pdf]

Brief FRIEDBEN Test Anxiety Scale

Directions: please mark the number that best describes your feeling in relation to an OSCE.

1. **1. If I fail a test, I am afraid what my friends will think.**
   Mark only one oval.

   - 1. Does not describe me at all
   - 2.
   - 3.
   - 4.
   - 5.
   - 6. Describes me perfectly

2. **2. If I fail a test, I am afraid people will consider me worthless.**
   Mark only one oval.

   - 1. Does not describe me at all
   - 2.
   - 3.
   - 4.
   - 5.
   - 6. Describes me perfectly

3. **3. I am very worried about what my teacher will think or do if I fail this test.**
   Mark only one oval.

   - 1. Does not describe me at all
   - 2.
   - 3.
   - 4.
   - 5.
   - 6. Describes me perfectly

4. **4. I am worried that all my friends will get high scores on the test and only I will get low ones.**

Mark only one oval.

- 1. Does not describe me at all
- 2.
- 3.
- 4.
- 5.
- 6. Describes me perfectly

5. **5. I am worried that failure on the test will embarrass me socially.**

Mark only one oval.

- 1. Does not describe me at all
- 2.
- 3.
- 4.
- 5.
- 6. Describes me perfectly

6. **6. During a test, my thoughts are clear and I answer all questions.**

Mark only one oval.

- 1. Describes me perfectly
- 2.
- 3.
- 4.
- 5.
- 6. Does not describe me at all

7. **7. During a test, I feel that I'm in good shape and that I'm organised.**

Mark only one oval.

- 1. Describes me perfectly
- 2.
- 3.
- 4.
- 5.
- 6. Does not describe me at all

8. **8. I feel that my chances are good to perform well on tests.**
Mark only one oval.

- 1. Describes me perfectly
- 2.
- 3.
- 4.
- 5.
- 6. Does not describe me at all

9. **9. I usually function well on tests.**
Mark only one oval.

- 1. Describes me perfectly
- 2.
- 3.
- 4.
- 5.
- 6. Does not describe me at all

10. **10. I am very tense before a test, even if I am well prepared.**
Mark only one oval.

- 1. Does not describe me at all
- 2.
- 3.
- 4.
- 5.
- 6. Describes me perfectly

11. **11. While I am taking an important test, my heart beats rapidly.**
Mark only one oval.

- 1. Does not describe me at all
- 2.
- 3.
- 4.
- 5.
- 6. Describes me perfectly

12. **12. I am terribly scared of tests.**

Mark only one oval.

- 1. Does not describe me at all
- 2.
- 3.
- 4.
- 5.
- 6. Describes me perfectly

Powered by
Google Forms
