# Supplementary material for: Evaluation of a coaching workshop for the management of veterinary nursing students’ OSCE-associated test anxiety
Source: Ir Vet J. 2018 Jul 27;71:15. doi: 10.1186/s13620-018-0127-z (PMC6064137; doi:10.1186/s13620-018-0127-z)
Supplement: Supplementary file 2 — mini-IPIP questionnaires. (PDF 79 kb) [file 13620_2018_127_MOESM2_ESM.pdf]

mini IPIP questionnaire

Please circle the option that best describes you with regards to each statement

1. **I talk to a lot of different people at parties**
   Mark only one oval.
   - Very inaccurate
   - Moderately inaccurate
   - Neither inaccurate nor accurate
   - Moderately accurate
   - Very accurate

2. **I get chores done right away**
   Mark only one oval.
   - Very inaccurate
   - Moderately inaccurate
   - Neither inaccurate nor accurate
   - Moderately accurate
   - Very accurate

3. **I have difficulty understanding abstract ideas**
   Mark only one oval.
   - Very inaccurate
   - Moderately inaccurate
   - Neither inaccurate nor accurate
   - Moderately accurate
   - Very accurate

4. **I sympathise with others' feelings**
   Mark only one oval.
   - Very inaccurate
   - Moderately inaccurate
   - Neither inaccurate nor accurate
   - Moderately accurate
   - Very accurate

5. **I am relaxed most of the time**
Mark only one oval.

- Very inaccurate
- Moderately inaccurate
- Neither inaccurate nor accurate
- Moderately accurate
- Very accurate

6. **I am the life of the party**
Mark only one oval.

- Very inaccurate
- Moderately inaccurate
- Neither inaccurate nor accurate
- Moderately accurate
- Very accurate

7. **I make a mess of things**
Mark only one oval.

- Very inaccurate
- Moderately inaccurate
- Neither inaccurate nor accurate
- Moderately accurate
- Very accurate

8. **I do not have a good imagination**
Mark only one oval.

- Very inaccurate
- Moderately inaccurate
- Neither inaccurate nor accurate
- Moderately accurate
- Very accurate

9. **I feel others’ emotions**
Mark only one oval.

- Very inaccurate
- Moderately inaccurate
- Neither inaccurate nor accurate
- Moderately accurate
- Very accurate

10. **I get upset easily**
Mark only one oval.

- Very inaccurate
- Moderately inaccurate
- Neither inaccurate nor accurate
- Moderately accurate
- Very accurate

11. **I don’t talk a lot**
Mark only one oval.

- Very inaccurate
- Moderately inaccurate
- Neither inaccurate nor accurate
- Moderately accurate
- Very accurate

12. **I often forget to put things back in their proper place**
Mark only one oval.

- Very inaccurate
- Moderately inaccurate
- Neither inaccurate nor accurate
- Moderately accurate
- Very accurate

13. **I am not interested in abstract ideas**
Mark only one oval.

- Very inaccurate
- Moderately inaccurate
- Neither inaccurate nor accurate
- Moderately accurate
- Very accurate

14. **I am not really interested in others**
Mark only one oval.

- Very inaccurate
- Moderately inaccurate
- Neither inaccurate nor accurate
- Moderately accurate
- Very accurate

15. **I have frequent mood swings**
Mark only one oval.

- Very inaccurate
- Moderately inaccurate
- Neither inaccurate nor accurate
- Moderately accurate
- Very accurate

16. **I keep in the background**
Mark only one oval.

- Very inaccurate
- Moderately inaccurate
- Neither inaccurate nor accurate
- Moderately accurate
- Very accurate

17. **I like order**
Mark only one oval.

- Very inaccurate
- Moderately inaccurate
- Neither inaccurate nor accurate
- Moderately accurate
- Very accurate

18. **I have a vivid imagination**
Mark only one oval.

- Very inaccurate
- Moderately inaccurate
- Neither inaccurate nor accurate
- Moderately accurate
- Very accurate

19. **I am not interested in other peoples' problems**
Mark only one oval.

- Very inaccurate
- Moderately inaccurate
- Neither inaccurate nor accurate
- Moderately accurate
- Very accurate

20. **I seldom feel blue**
Mark only one oval.

- Very inaccurate
- Moderately inaccurate
- Neither inaccurate nor accurate
- Moderately accurate
- Very accurate

Powered by
Google Forms
