# Supplementary material for: Evaluation of a coaching workshop for the management of veterinary nursing students’ OSCE-associated test anxiety
Source: Ir Vet J. 2018 Jul 27;71:15. doi: 10.1186/s13620-018-0127-z (PMC6064137; doi:10.1186/s13620-018-0127-z)
Supplement: Supplementary file 3 — focus group questions. (PDF 37 kb) [file 13620_2018_127_MOESM3_ESM.pdf]

Focus group questions

So would you like to share how your practice OSCE went?

Did the workshop have any effect on how you felt during the practice OSCE? Tell me about that.

Did the workshop have any effect on your performance during the practice OSCE? Tell me about that.

How did you feel while in the waiting area before starting the practice OSCE?

Did you listen to the music while waiting to start the OSCE? If no, why not? If yes, what did you think of it?

What other measures could help students before and during an OSCE?

Is there anything else you would like to add?
